# Enhancement or Suppression of ACE Inhibitory Activity by a Mixture of Tea and Foods for Specified Health Uses (FOSHU) That Are Marketed as “Support for Normal Blood Pressure”

**DOI:** 10.5402/2011/712196

**Published:** 2011-08-04

**Authors:** Isao Murakami, Hiroyuki Hosono, Shigeto Suzuki, Junichi Kurihara, Fumio Itagaki, Machiko Watanabe

**Affiliations:** ^1^Division of Medical and Pharmaceutical Sciences-II, Faculty of Pharmaceutical Sciences, Teikyo University, 2-11-1, Kaga, Itabashi, Tokyo 173-8605, Japan; ^2^Division of Medical and Pharmaceutical Sciences-I, Faculty of Pharmaceutical Sciences, Teikyo University, 1091-1, Suarashi, Midori, Sagamihara, Kanagawa 252-0195, Japan

## Abstract

The ACE inhibitory activities of mixtures of FOSHUs (*Healthya, Goma-Mugicha, Lapis Support* and *Ameal*) were examined in order to identify any antihypertensive interactions. Among combinations of *Healthya* with other samples that contain active peptides, only that with *Ameal* was found to have no inhibitory activity. Enhanced activity was observed in 2 other mixtures. The activity of a mixture of tea polyphenols and the whey component extracted from an *Ameal* solution was significantly decreased, thus demonstrating that whey protein lowered the ACE inhibitory activity of *Healthya*. Although oral administration of tea polyphenols alone significantly decreased SBP in SHR at 2 and 4 hr, combined administration with *Ameal* failed to decrease SBP at the same time points. In conclusion, the simultaneous intake of tea and FOSHUs that contain active peptides might affect daily self-antihypertensive management via enhancement or suppression of ACE inhibitory activity.

## 1. Introduction

Tea polyphenols contained in green tea leaves (*Camellia sinensis L.*) are known to have antiviral [1], antioxidative [2, 3], antimutagenic [4], anticarcinogenic [5], antiobesity [6], and antihypertensive activities [7–9]. Tea leaves contain tea polyphenols such as catechins, so regular consumption of green tea is thought to be beneficial to one's health.

With rising health consciousness among consumers, particular attention has recently focused on green tea's action to counter obesity and hypertension, which are diagnostic criteria for metabolic syndrome [10]. Tea beverages that contain high levels of tea polyphenols and that are labeled “For individuals worried about body fat” are being sold in Japan as foods for specified health uses (FOSHUs).

Long-term consumption of tea has been reported to result in antihypertensive activity in spontaneously hypertensive rats (SHRs) [7, 8] while another study reported antihypertensive activity with a single administration of tea [9]. The antihypertensive activity of tea polyphenols is thought to occur via the inhibition of angiotensin-converting enzyme (ACE) activity [11]. Moreover, other FOSHUs, many of which have active components consisting of peptides that inhibit ACE activity, are marketed to target “individuals with high blood pressure” [12–14].

Indeed, tea beverages and FOSHUs can be readily obtained, so there may be instances in which they are consumed together. However, for individuals with high blood pressure, little is known about the antihypertensive interaction between tea and FOSHUs, which are both thought to have the same mechanisms of action.

Thus, the aim of the current study was to examine the antihypertensive interaction when a tea beverage and a FOSHU “For individuals with high blood pressure” (*Goma-Mugicha*, *Lapis Support*, and *Ameal*) were consumed together.

## 2. Materials and Methods

### 2.1. Materials

The green tea beverage used was *Healthya Green Tea* (Kao Corporation, Tokyo). The 3 FOSHU products were *Ameal* (Calpis Co., Tokyo), which is claimed to “help maintain a normal blood pressure level” and contains Val-Pro-Pro as its active ingredient [12], *Lapis Support* (Tokiwa Yakuhin Co., Ltd., Yamaguchi, Japan), which has Val-Tys as its active ingredient [13], and *Goma-Mugicha* (Suntory Foods Ltd., Tokyo), which has Leu-Val-Tys as its active ingredient [14]. Whey protein concentrate (WPC: *Protein*, Easy Sports Co., Ltd., Tokyo) was also used. *Polyphenon CG* (green tea extract, tea polyphenol content of at least 30%) was purchased from Mitsui Norin Co., Ltd. (Tokyo). Rabbit lung ACE was purchased from Sigma Chemical Co. (St. Louis, MO). All other chemicals were obtained from Wako Pure Chemicals Co. (Tokyo).

### 2.2. Preparation of Ameal

The whey fraction was used to determine the ACE inhibitory activity of *Ameal*. The whey fraction was obtained as follows [15]. The pH of *Ameal *was adjusted to 3.4 by adding 50% lactic acid. The *Ameal* was centrifuged at 6,000 × g for 10 min, 10 N NaOH was added to the supernatant to raise the pH to 8.3, and then the supernatant was centrifuged at 6,000 × g for 10 min. The whey fraction of *Ameal *was used as a sample.

### 2.3. Assay of ACE Inhibitory Activity

ACE inhibitory activity was assayed according to the method of Suzuki et al. [16] with some modifications. Hippuryl-L-histidyl-L-leucine (Hip-His-Leu) was dissolved in 400 mM phosphate buffer (pH 8.5) containing 300 mM NaCl. Next, 0.1 mL of 4.7 mM Hip-His-Leu solution was mixed with 0.05 mL of sample solution (*Healthya*,* Goma-Mugicha*,* Lapis Support*, *Ameal*, and their mixtures) and then preincubated for 5 min at 37°C. The reaction was initiated by the addition of 0.1 mL of ACE dissolved in distilled water (25 mU/mL), and the mixture was incubated for 30 min at 37°C. After the reaction had been stopped by adding 0.3 N NaOH (0.75 mL), 0.05 mL of 2% *o*-phthaldialdehyde in methanol was added, the mixture was left at room temperature for 10 min, and then 0.1 mL of 0.1 N HCl was added. After incubation for 30 min at room temperature, the amount of liberated His-Leu was determined by measuring the fluorescent intensity of its adduct with *o*-phthaldialdehyde (excitation at 340 nm and emission at 455 nm). The extent of inhibitory activity was calculated as follows:

(1) $$100-\left[\frac{B-A}{B-C}\times 100\right],$$

where *A* is the fluorescent intensity in the presence of ACE and ACE inhibitory component, *B* is the fluorescent intensity without ACE inhibitory component, and *C* is the fluorescent intensity without ACE. 

Inhibition was expressed as the concentration of component that inhibits 50% of ACE activity (IC_50_). One unit of ACE inhibitory activity was expressed as the potency showing 50% ACE inhibition under these conditions and was calculated for one bottle, daily intake, or maximum dosage.

### 2.4. Measurement of *β*-Lactoglobulin Concentrations

The concentration of *β*-lactoglobulin (*β*-Lg) was measured using a FASPEK milk (*β*-lactoglobulin) kit (Morinaga Institute of Biological Science, Yokohama, Japan) [17].

### 2.5. Animals

Male SHRs (SHR/NCrlCrlj) were purchased from Charles River Japan (Yokohama). The SHRs were kept in an air-conditioned room at 22–24°C with 55% humidity and a 12 hr light-dark cycle (lights on at 8:00 AM). Food and water were freely available. After an overnight fast, 2 g of *Polyphenon CG* were dissolved in distilled water or 6 mL of *Ameal* and orally administered at a dose of 6 mL/kg to conscious SHRs (body weight: 320–390 g, 22–25 weeks old) using a feeding needle. Following administration, systolic blood pressure (SBP) was measured every 2 hr. SBP after administration was measured by the tail-cuff method using an MK-2000 blood pressure monitor (Muromachi Kikai Ltd., Japan). The studies were performed according to the guidelines of the Animal Care and Use Committee of Teikyo University. All experiments conformed to the guidelines on the ethical use of animals, and all efforts were made to minimize both the number of animals used in the experiments and their suffering.

### 2.6. Statistical Analysis

Results are expressed as the mean and standard deviation (SD). The significance of differences in ACE inhibitory activity and SBP was analyzed using the Dunnett test or Tukey test.

## 3. Results and Discussion

### 3.1. Effects of Several Mixtures of Tea and FOSHU on ACE Inhibitory Activity

**Figure 1:**
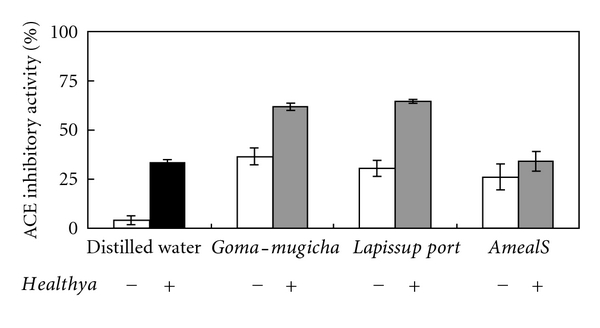
ACE inhibitory activity of mixtures of *Healthya* and FOSHU. Activity of *Healthya* with respect to converting 32.5 mg/mL of tea polyphenols (black square) and ACE inhibitory peptides in a FOSHU (white square: *Goma-Mugicha* conversion of 10 ng/mL Leu-Val-Tys, *Lapis Support *conversion of 38 ng/mL Val-Tys, and *Ameal *conversion of 445 ng/mL Val-Pro-Pro) or a mixture of *Healthya* and a FOSHU (gray square). Data are expressed as mean ± SD, *n* = 3.

ACE inhibitory activity (IC_50_) was detected in all samples (64.8 × 10^3^ ± 7.3 × 10^3^ ng/mL, 19.36 ± 4.87 ng/mL, 76.67 ± 11.12 ng/mL, and 882.70 ± 72.70 ng/mL, resp.) (Table 1). When *Healthya* was mixed with one of the FOSHUs, the two mixtures of *Healthya* plus *Goma-Mugicha* and *Healthya* plus *Lapis Support* had enhanced inhibitory activity. The mixture of *Healthya *plus *Ameal* did not have enhanced inhibitory activity. The ACE inhibitory activity shown in Figure 1 was calculated as IC_50_ based on the amount of functional components included in each food product for specified health uses.

### 3.2. Effects of a Mixture of Tea and Whey Protein on ACE Inhibitory Activity

**Figure 2:**
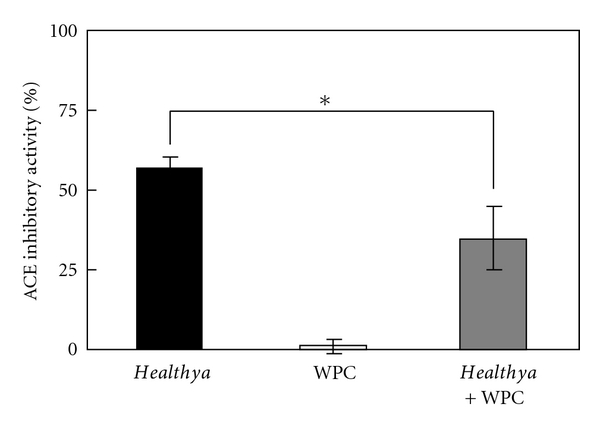
ACE inhibitory activity of *Healthya* with or without WPC. Activity of *Healthya* with respect to converting 65 mg/mL of tea polyphenols (black square), 3 mg/mL WPC (white square), or a mixture of *Healthya* and WPC (gray square). Data are expressed as mean ± SD, *n* = 3. (*P < 0.05, Tukey test).

In order to determine why the ACE inhibitory activity of a mixture of *Healthya* and *Ameal* was not enhanced, the elemental composition of each FOSHU was investigated. The whey fraction used in this experiment was in fact extracted from an *Ameal* solution (*Goma-Mugicha* and *Lapis Support* do not contain any ingredients that originate from milk). *Healthya* that was prepared to determine the IC_50_ was mixed with 3 *μ*g/mL WPC, and the mixture's rate of ACE inhibitory activity was measured. The concentration of *β*-lactoglobulin, the main component of whey protein [18], was 1.28 *μ*g/mL in 3 *μ*g/mL WPC and 18.6 *μ*g/mL in the whey fraction of *Ameal* (concentration of IC_50_, 445 ng/mL Val-Pro-Pro). WPC (3 *μ*g/mL, as whey) and *Healthya* (as tea polyphenol) were used for measurement. Examination of the ACE inhibitory activity of a mixture of whey component and tea polyphenols revealed a significant decrease in the rate of ACE inhibitory activity of *Healthya* (56.9 ± 3.2%) as a result of the addition of 3 *μ*g/mL WPC (34.9 ± 9.8%: *P* < 0.05) (Figure 2), thus demonstrating that whey protein lowered the rate of ACE inhibitory activity of* Healthya.*

### 3.3. Effects of Tea Polyphenols and Ameal on Antihypertensive Activity in SHRs

**Figure 3:**
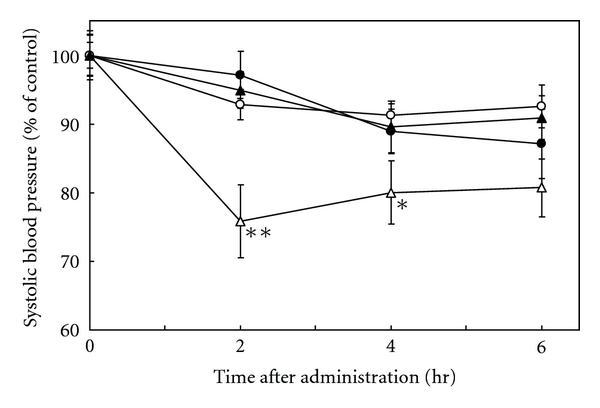
Time course of hypotensive activity after oral administration of tea polyphenols (*Polyphenon CG*) to SHRs. *⚫*, Distilled water (control); ○, *Ameal*; ▲, *Polyphenon CG* in *Ameal*; △, *Polyphenon CG* in distilled water. The vertical axis represents changes in SBP from time zero. Changes in SBP from time zero are expressed as the mean ± SD, *n* = 7. The baseline SBP before administration was 198.2 ± 3.0 mmHg (*P < 0.05, **P < 0.01 cosmpared to distilled water, Dunnett's test).

To evaluate the antihypertensive activity of the interaction of tea polyphenols and *Ameal*, the SBP of SHRs was measured after a single oral administration. The baseline SBP before administration was 198.2 ± 3.0 mmHg. Tea polyphenols (*polyphenon CG*, 2 g/kg body weight) significantly lowered SBP 2 and 4 hr after administration (155.4 ± 8.4 mmHg and 163.8 ± 7.6 mmHg, resp.) in comparison to distilled water (195.3 ± 6.7 mmHg and 178.8 ± 5.8 mmHg, resp.) (*P* < 0.05). However, oral administration of *polyphenon CG* and *Ameal *failed to decrease SBP (178.9 ± 4.7 mmHg and 169.2 ± 8.3 mmHg, resp.) at the same times (Figure 3). Therefore, combined administration of *Polyphenon CG* with *Ameal* appears to attenuate antihypertensive activity by interacting with the whey protein in the *Ameal* solution. Though possible mechanisms of the interaction of tea and milk protein have been discussed in previous reports [19–21], the actual mechanism is still not clear 

In this study, markedly larger amounts of tea catechins than humans would normally be consumed in typical foods that were administered to SHRs, and this study examined the interaction of these catechins. While many health foods contain much smaller amounts of substances than were used in the current experiments, numerous products are being sold in formulations with active components in higher concentrations than that would normally be consumed. In addition, the amount of health foods consumed is substantial when individuals have a strong predilection toward self-medication. Many such individuals will presumably consume numerous types of health foods at the same time. Accordingly, large quantities of tea catechins were used in the current study, and the consumption of such amounts is indeed a possibility.

Moreover, this study focused on blood pressure, which was found to be affected soon after administration [9], but interaction between tea catechins and whey protein attenuated the activity of the two, much like other types of activity (e.g., antiviral, antioxidative, antimutagenic and anticarcinogenic, and antiobesity activity) are attenuated. Based on results of the current study, consuming multiple health foods at the same time enhanced the activity of individual components in some instances. That said, the results also suggested that such consumption led to interactions, diminishing the effectiveness of some of the components of these foods.

## 4. Conclusion

The findings of the present study suggest for the first time that interaction between tea and milk protein may attenuate antihypertensive activity and that the simultaneous intake of tea and FOSHUs that contain active peptides might affect daily self-antihypertensive management via the enhancement or suppression of ACE inhibitory activity.

## Figures and Tables

**Table 1:** ACE inhibitory activity of green tea beverage and FOSHU. ACE inhibitory activity is shown as mean ± SD, *n* = 6. *The ACE inhibitory activity is shown in relation to the concentration of tea polyphenol included in *Healthya *or functional component in each FOSHU.

Sample	ACE inhibitory activity IC_50_ (ng/mL)*
*Healthya*	64.8 × 10^3^ ± 7.3 × 10^3^
*Goma-Mugicha*	19.36 ± 4.87
*Lapis Support*	76.67 ± 11.12
*Ameal*	882.70 ± 72.70
